# Strategies for the Management of Aggressive Invasive Plant Species

**DOI:** 10.3390/plants12132482

**Published:** 2023-06-28

**Authors:** Paula Lorenzo, Maria Cristina Morais

**Affiliations:** 1 University of Coimbra, Department of Life Sciences, Centre for Functional Ecology (CFE)—Science for People & the Planet, TERRA Associate Laboratory, 3000-456 Coimbra, Portugal; 2 Centre for the Research and Technology of Agro-Environmental and Biological Sciences (CITAB), Inov4Agro, Institute for Innovation, Capacity Building and Sustainability of Agri-Food Production, University of Trás-of-Montes and Alto Douro, Quinta de Prados, 5000-801 Vila Real, Portugal; cmorais@utad.pt

**Keywords:** invasive alien plants, waste from invasive plants, waste use, alternative strategies, sustainable long-term management

## Abstract

Current control methods for invasive alien plants (IAPs) have acceptable short-term outcomes but have proven to be unfeasible or unaffordable in the long-term or for large invaded areas. For these reasons, there is an urgent need to develop sustainable approaches to control or restrict the spread of aggressive IAPs. The use of waste derived from IAP control actions could contribute to motivating the long-term management and preservation of local biodiversity while promoting some economic returns for stakeholders. However, this strategy may raise some concerns that should be carefully addressed before its implementation. In this article, we summarize the most common methods to control IAPs, explaining their viability and limitations. We also compile the potential applications of IAP residues and discuss the risks and opportunities associated with this strategy.

## 1. Introduction

Scientific studies over the last few decades have highlighted invasive alien plants (IAPs) as one of the major threats to ecosystems [1,2,3], especially those growing in protected areas [4,5]. Plant invasions are mainly influenced by direct (e.g., by transport propagules) or indirect (e.g., by altering land use) human actions that involve moving plants around the world for different purposes [6]. For example, many exotic plant species were planted to provide products and benefits that support livelihoods [7]. Once established in a new region, some exotic plants rapidly expand and become invasive, causing significant losses in biodiversity, ecosystem functioning and services, socio-economic values, and human health in the invaded areas [8]. Invasive alien plant species have strong adaptability, fast reproduction, spreading capabilities, and other traits that contribute to their success in their new area. Climate change leading to environmental constraints can also increase opportunities for the establishment of IAPs, which are better able to acquire limited resources or use resources more efficiently than native plant species [9].

Invasive alien plant species have colonized almost all types of terrestrial ecosystems and aquatic environments worldwide, except for Polar biomes. For example, *Acacia* spp. in Mediterranean areas [1], *Prosopis* spp. in arid environments [10], *Carpobrotus* spp. in coastal areas [11], *Robinia pseudoacacia* L. and *Ailanthus altissima* (Mill.) Swingle in mixed forests [12], *Oxalis pes-caprae* L. in ruderal and agricultural lands [13,14], and *Eichhornia crassipes* (Mart.) Solms in water bodies and courses [15,16]. Invasive plants can form homogeneous stands that have significant impacts on both the above and belowground compartments of invaded ecosystems [17,18,19]. These impacts include changes in resident plant communities and soil microbes [17,18,20,21], animal populations [22,23], soil properties and nutrient cycling [24,25], fire regimes and water flow [2,23,26], and biotic interactions [27,28]. In addition, IAPs can threaten human health and the socio-economy of the region [29]. However, the economic costs associated with the detrimental impacts of IAPs remain largely unknown. According to the most recent database of invasion costs (InvaCost), the economic costs of the biological invasion reached USD 1.22 trillion in the USA [30] and EUR 116.61 billion in Europe [31] between 1960 and 2020, with a clear dominance of damage costs over management expenditure [32], focusing on eradication and control actions [33]. In the Mediterranean Basin as well as in Europe, *Ambrosia artimisiifolia* L. is the costliest IAP [31,34]. Moreover, the future overall invasion costs are expected to increase, which emphasizes the urgent need to allocate more resources for managing invasive species.

Here, we aim to (i) compile current methods used for controlling and managing IAPs, enumerating their viability and main weaknesses, (ii) present potential applications of IAP waste, and (iii) discuss the risks and opportunities associated with implementing a strategy for using IAP waste which could help to reduce the costs associated with their control.

## 2. Current Methods to Control Invasive Plants, Their Viability, and Problems

Some IAPs can have positive effects in several areas, including agriculture, the ornamental horticulture industry, and wood production, but their use can result in harmful effects, representing a conflict of interest in their management [35,36]. Harmful IAPs need to be controlled due to the magnitude of their environmental, economic, social, and aesthetic impacts. In dramatic cases of invasion, when IAPs show high vegetative reproduction and are widely distributed across large areas, eradication is extremely difficult and expensive. Therefore, confining invasive populations and limiting their spread through effective strategies should be prioritized.

Traditional management methods for terrestrial and aquatic IAPs include physical (manual or mechanical) methods such as pulling and digging, debarking, harvesting [37], mowing and tilling [38], prescribed fire [39], soil solarization [40], construction of barriers to limit the spread of aquatic weeds [41], usage of chemical (e.g., herbicide application [37,42,43]), biological control [37,44], or a combination of several methods [45,46]. The feasibility of each method depends on the species, the extent of invasion, the characteristics of the habitat, and the effectiveness of the control methods [47]. A recent Australian study based on the stakeholders’ perspective in the management of IAPs noted that decision-makers are more confident in the use of chemicals than biocontrol and mechanical methods in the control of IAPs and believe that it is more feasible to control succulents and herbs/shrubs than monocots and woody vines [48].

Although the existing control methods often produce relatively good short-term outcomes, they are ineffective in eradicating IAPs in the long-term if not applied periodically [3,49,50]. For example, a recent study by Froeschlin et al. [51] in South Africa, a pioneering country in managing IAPs, noted that after 10 years of adopting a combination of control measures (mulching, herbicides, and the sowing of native species) to restore invaded areas, IAPs were not totally extirpated, indicating the need to improve techniques and implement additional efforts to eliminate them. Also, Duarte et al. [52] stressed the importance of frequent follow-up actions to reduce the abundance of *Acacia longifolia* (Andrews) Willd. in coastal dunes. Success in containing the dispersion or ultimately eradicating IAPs requires consistent post-surveillance and follow-up actions within an integrated strategy framework, which represents a huge challenge in terms of the available budget and execution timeframe [53]. In the absence of ongoing management, escaped individuals from the management can act as new focal dispersal points [51], leading to potential recolonization of the area and a loss of previous control efforts. However, the long-term management of IAPs is often neglected in part due to unaffordable costs for sustained efforts or a lack of interest after controlling the initial target population. Hence, it is necessary to adopt a more economical and efficient strategy to manage IAPs, for example, by deriving benefits from the management of plant invasion.

Besides economic problems, traditional methods also lead to social and environmental side effects. The use of synthetic herbicides can negatively affect human health, including neurological, reproductive, and respiratory diseases, diabetes, and even cancer [54], and the environment by reaching non-target organisms [55], raising concerns about their use. The repeated use of chemicals may also lead to invasive populations developing resistance to herbicides [56]. On the other hand, the introduction of generalist biological control agents can infect non-target species, causing a decline in their populations [57]. Another drawback common to most traditional control methods and anticipated in the previous paragraphs is the poor cost-effectiveness of the relationship between the invader spreading and the controlled area or the eradication time. As an example, the eradication of *Alliaria petiolata* (Bieb.) Cavara and Grande from an area of Adirondack Park (United States) was predicted to take 11 years with 100% effective control or more than 50 years with only 90% effective control [58]. In general, the management of IAPs is very expensive [31], with control strategies still prioritizing key landscape points to disrupt invasion connectivity and likely reduce costs without covering the whole area [59]. Even the long-term and well-designed South African program “Working for Water” is far from controlling the entire estimated invaded area [60]. Such limitations with frequent uncertain outcomes make traditional control methods unsafe, unfeasible, or unaffordable for large areas.

Recently, proactive strategies such as avoiding introduction, early detection, and rapid intervention (Regulation (EU) No 1143/2014 of the European Parliament and of the Council of 22 October 2014), prioritization of control actions, and citizen awareness have been proposed as key to prevent the spread of IAPs and reduce their negative impacts and control costs [37,61,62,63]. Involving volunteers is also an important component of monitoring and controlling IAPs [64], helping to reduce costs while increasing public awareness [65]. However, this solution is impractical for the rapid control of infested or heavily invaded areas since it is necessary for there to be continuous engagement and training of the volunteers [66]. Therefore, designing cooperative strategies in biosecurity among affected countries [36] is essential for the development of more cost-effective actions against IAPs. Despite these recent strategies preventing the introduction of new exotics, which are totally necessary and welcome, they are still in their infancy and do not greatly improve the control of IAPs already established. For these reasons, it is crucial to find alternative strategies to control current aggressive IAPs in a more sustainable way.

## 3. Potential Applications of IAP Waste from Management Actions

**Figure 1:**
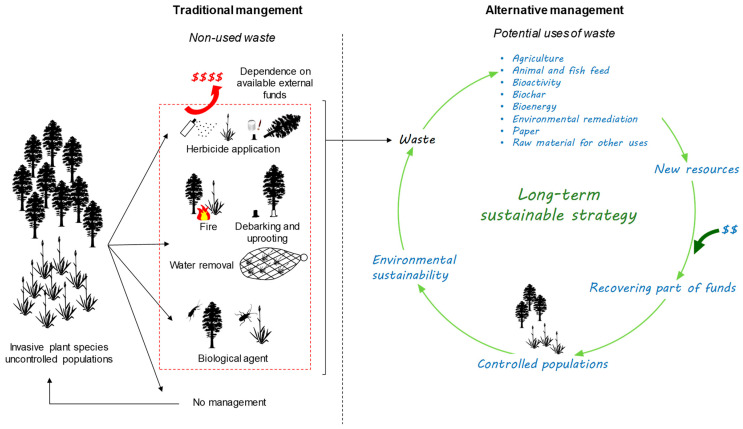
Scheme for the proposed management for the control of highly invasive alien plants.

The use of waste generated by the removal of aggressive IAPs, with the objective of reducing the large costs associated with the control of these species, is a viable alternative. The use of invasive waste can provide novel value-added products, resulting in not only profits for society but also helping to preserve local biodiversity and stimulate the long-term management of invaded areas [67]. With this approach, it would be possible to partially recover invested funds that would otherwise be lost [42,67,68]. Moreover, it prevents the use of limited resources by introducing underused material into the system, which aligns with the circular bioeconomy rationale (Figure 1) and Sustainable Development Goals 12 (responsible consumption and production) and 15 (life on land) of the 2030 Agenda.

The waste from IAPs exhibits specific properties (functional, biological, medicinal, etc.) that can be used directly or serve as a basis for new products, as summarized in Table 1. For example, waste biomass, especially that from invasive legumes, but not only them, is primarily a source of nutrients that can be used as fertilizers, horticultural substrates, or soil amendments in agriculture either directly or after a composting process [69,70,71,72,73,74,75,76,77,78,79,80,81,82,83,84,85,86,87,88,89,90,91,92,93,94,95,96,97,98,99,100,101,102,103]. Invasive plants with allelopathic/phytotoxic effects, such as *Acacia dealbata* Link, *Solidago canadensis* L., or *S. gigantea* Aiton, can provide compounds with pesticidal or biostimulant effects appropriate for agricultural purposes [104,105,106,107]. Some IAPs are also a source of bioactive compounds with beneficial antioxidant, antimicrobial, nutraceutical, pharmacological, cosmetic, or therapeutical-related applications (e.g., [16,108,109,110,111,112,113,114,115,116,117,118,119,120,121,122,123,124,125,126,127,128,129,130,131,132]). Invasive waste can also be used to produce bioenergy, namely bioethanol, biogas, or wood fuel (e.g., [133,134,135,136,137,138,139,140,141,142,143,144,145,146,147,148]), biochar or charcoal for different purposes (e.g., [149,150,151,152,153]), or animal feed (e.g., [154,155,156,157]. Some authors also suggest the use of IAPs for effluent treatments (e.g., [158,159,160,161,162,163,164,165]), paper and packaging materials (e.g., [166,167,168,169,170]), building materials [171], natural fiber composites [172], and bio-adsorbents for textile dyes or others [173,174,175,176,177,178,179,180].

## 4. Remarks on the Risks and Opportunities of Adopting the Use of Waste from IAPs

The use of waste from IAPs to control invasive populations, although not completely new, is advocated by several authors, as mentioned in Table 1 [92,126]. This idea is not broadly accepted among plant invasion ecologists [181], who argue that it can promote the cultivation of IAPs or facilitate their spread due to negligence in the treatment/transport of waste. This concern is valid given that plant invasion is a sensitive issue, and using IAPs poses the risk of incentivizing its growth instead of its control [182]. However, when invasion achieves a dramatic level and traditional control methods prove to be physically or economically insufficient or fail over time, which is associated with the inability to conduct follow-up actions after the end of a management project, the use of IAP waste could be viewed as a sustainable and viable solution to control and restrict the expansion of aggressive IAPs (Figure 1). The control of widespread IAPs, such as Australian acacias (*Acacia* spp.), water hyacinth (*E. crassipes*), or pampas grass (*Cortaderia selloana* (Schult. and Schult.f.) Asch. and Graebn.) [9,20,24,183], which have high vegetative reproduction and/or produce huge quantities of long-lived seeds that make their control difficult even after clearing an invaded area [67], generates large quantities of waste, often left on-site or burned for energy purposes [184]. The abandonment of the plant material may be a fire hazard [2,185], which can also contribute to the death of native plants and increase the risk of re-invasion [2], resulting, in turn, in the loss of control action benefits. In these situations, we suggest that using the waste from control actions is a reasonable approach to manage the overabundance of these IAPs in the long-term. The implementation of this approach may reduce the continuous spread of IAPs and contribute to the partial recovery of management funds, creating conditions for the sustainability of the process (Figure 1). In addition, the conversion of waste biomass from IAPs into new, useful products can promote zero-waste and circular economy approaches [186]. The potential uses of waste derived from IAPs have gained increasing interest in the last years, as summarized in Table 1. The majority of these studies were performed on a laboratory scale and focused on the potential of IAPs for further investigation and application in different areas, but relatively few studies include a cost–benefit analysis of using waste from IAPs. The work of Mudavanhu et al. [187] is one of the studies that discuss the economic implications of using the waste of *Acacia cyclops* A. Cunn. ex Don fil. for electricity generation in South Africa and concluded that it is a viable and feasible option in comparison with electricity production by diesel generators. On the contrary, Melane et al. [188], also in South Africa, observed that the costs of using biomass of seven non-woody IAPs, namely *Arundo donax* L., *Lantana camara* L., *Solanum mauritianum* Scop., *Atriplex nummularia* Lindl., *Cestrum laevigatum* Schlecht, *Senna didymobotrya* (Fresen.) H. S. Irwin and Barneby, and *Chromoleana odorata* (L.) R.M.King and H.Rob, were very high when compared to woody IAPs, suggesting that these IAPs are not a profitable resource for the production of electricity. Another study conducted by Valen [189] with the aim of investigating the cost–benefits of *Arundo donax* as feedstock for the pulp and paper industry in California, indicated an average annual profit of ca. 60% of removal expenses, which is an encouraging indicator of the financial viability of the process. As indicated by Ortega et al. [172], it is important to emphasize that the economic benefits achieved by using IAP biomass should be only to minimize the costs of their management and control and ultimately contribute to the reduction of the area occupied by these species. However, complete economic analyses assessing all aspects of using IAP waste, including management actions, waste transport, waste processing, and the benefit of the final product, are still lacking.

The use of invasive waste should be adopted within a clear regulatory framework to avoid bias in the utilization of this waste [182]. As stated before, the cultivation of IAPs is legally prohibited (Regulation (EU) No 1143/2014 of the European Parliament and of the Council of 22 October 2014), but the use of IAP waste derived from control actions lacks legal policies. To ensure that this strategy is properly applied, it is a prerequisite that a long-term management plan for IAPs is applied. In addition, it is necessary to select a control method (please see Section 2) that minimizes the risk of re-invasion and implement a monitoring plan for the controlled area after management actions. A set of recommendations for control, transport, and waste disposal after the control takes place should also be considered to avoid the dispersion of new propagules and the re-establishment of the invader. For example, control actions should take place when plants have no flowers or fruits/seeds to prevent seed dispersal. The location of disposal sites should be in areas with minimal possibility of IAP establishment, and the area should be monitored to prevent their spread. For IAPs that reproduce by seeds (e.g., *Acacia* spp. and *Cortaderia selloana*), the plant material should be left on site to dry. For IAPs that present viable (root or stem) fragments (e.g., *Carpobrotus edulis* and *Eichhornia crassipes*), the plant material should be left on site with the roots upward to prevent them from contacting the soil. In both situations, transport should occur when all plant material is completely dry, and the operation should be monitored for the presence of any type of viable structures.

In summary, the use of waste from IAPs is a strategy that feeds on and completes traditional control actions to facilitate the management of aggressive IAPs. However, this strategy should only be conducted in specific and dramatic invasion cases, when traditional control methods have failed over time, and should be conducted under strict regulations. This strategy should be accompanied by a complete economic analysis covering the entire IAP management and use of the waste process.

## Figures and Tables

**Table 1:** Reported uses for invasive plant species.

Potential Use	Invasive Plant Species	Reference
Agriculture-related uses (fertilizers, compost, vermicompost, bioherbicides, etc.)	*Acacia dealbata* Link, *Acacia longifolia* (Andr.) Willd., *Acacia melanoxylon* R.Br., *Acacia podalyriifolia* A.Cunn. ex G.Don, *Acacia* spp., *Ailanthus altissima* (Mill.) Swingle, *Ageratina adenophora* (=*Eupatorium adenophorum*) (Spreng.) R.M.King and H.Rob., *Albizia julibrissin* Durazz., *Azolla filiculoides* Lam., *Eichhornia crassipes* (Mart.) Solms, *Fallopia japonica* (=*Reynoutria japonica*) (Houtt.) Ronse Decr., *Hedychium gardnerianum* Sheppard ex Ker Gawl., *Ipomoea staphylina* Roem. and Schult., *Lespedeza cuneata* (Dum.Cours.) G.Don, *Litsea glutinosa* (Lour.) C.B.Rob., *Myriophyllum* spp., *Parthenium hysterophorus* L., *Prosopis juliflora* (Sw.) DC., *Tithonia diversifolia* (Hemsl.) A.Gray, *Typha latifolia* L.	[69,70,71,72,73,74,75,76,77,78,79,80,81,82,83,84,85,86,87,88,89,90,91,92,93,94,95,96,97,98,99,100,101,102,103,104,105,106,107]
Bioactivity (pharmaceutical, nutraceutical, cosmetic, etc.)	*A. dealbata*, *A. melanoxylon*, *A. adenophora* (=*E. adenophorum*), *A. altissima*, *Amaranthus retroflexus* L., *Calotropis procera* (Aiton) A.W. Aiton, *Disphania ambrosioides* (L.) Mosyakin and Clemonts, *Dittrichia graveolens* (L.) Greuter, *E. crassipes*, *Erigeron annuus* (L.) Pers., *F. japonica* (=*R. japonica*), *Gleditsia triacanthos* L., *Heracleum mantegazzianum* Sommier and Levier, *Polygonum cuspidatum* Siebold. and Zucc., *Solidago canadensis* L., *Solidago gigantea* Aiton, *Spartina anglica* C.E.Hubb., *Tradescantia fluminensis* Vell., *Ulex europaeus* L.	[16,81,87,88,92,104,108,109,110,111,112,113,114,115,116,117,118,119,120,121,122,123,124,125,126,127,128,129,130,131,132]
Bioenergy (bioethanol, biogas, wood fuel, etc.)	*A. dealbata*, *A. adenophora* (=*E. adenophorum*), *A. altissima*, *C. procera*, *Dioscorea bulbifera* L., *E. crassipes*, *Eucalyptus globulus* Labill, *F. japonica* (=*R. japonica*), *H. mantegazzianum*, *Impatiens glandulifera* Royle, *Limnocharis flava* (L.) Buchenau, *Phragmites australis* (Cav.) Trin. Ex Steud., *Phalaris arundinacea* L., *Schinus terebinthifolius* Raddi, *S. gigantea*, *Spartina alterniflora* Loisel., *Typha* spp.	[81,83,84,85,86,87,88,133,134,135,136,137,138,139,140,141,142,143,144,145,146,147,148]
Biochar (activated carbon and biochar precursors)	*A. dealbata*, *Acacia auriculiformis* A.Cunn. ex Benth., *A. adenophora* (=*E. adenophorum*), *A. altissima*, *Alternanthera philoxeroides* (Mart.) Griseb., *D. bulbifera*, *E. crassipes*, *Leucaena leucocephala* (Lam.) de Wit, *Mimosa pigra* L., *P. juliflora*, *S. terebinthifolius*, *S. canadensis*, *S. gigantea*, *S. alterniflora*	[81,87,88,142,149,150,151,152,153]
Feed (animal or fish)	*A. altissima*, *C. procera*, *Cyperus* spp., *E. crassipes*, *H. gardnerianum*, *Hottonia* spp., *Lemna minor* (L). Griff, *Leucaena* spp., *Myriophyllum* spp., *Nasturtium* spp., *Pistia stratiotes* L., *Pittosporum undulatum* Vent., *P. juliflora*, *S. alterniflora*, *Typha* spp.	[81,85,86,87,94,154,155,156,157]
Environmental remediation (wastewater treatment, etc.)	*A. dealbata*, *A. altissima*, *C. procera*, *E. crassipes*, *Myriophyllum* spp., *P. arundinacea*, *Phragmites australis* (Cav.) Trin. ex Steud., *Pistia stratiotes* L., *Typha* spp.	[83,85,86,93,94,115,146,158,159,160,161,162,163,164,165]
Paper-related uses (handmade, etc.)	*A. altissima*, *E. crassipes*, *Fallopia x bohemica* (J.Chrtek and A.Chrtková) J.P. Bailey, *F. japonica* (=*R. japonica*), *Fallopia sachalinensis* (C.F. Schmidt) Ronse Decr., *S. canadensis*, *S. gigantea*, *Robinia pseudoacacia* L., *Rudbeckia laciniata* L.	[15,78,87,166,167,168,169,170]
Raw materials to produce different products (biorefineries, nanoparticles, resins, crafts, building materials, eco-bases, textile dyeing, etc.)	*A. dealbata*, *A. longifolia*, *Arundo donax* L., *C. procera*, *E. crassipes*, *F. japonica* (=*R. japonica*), *Mikania micrantha* Kunth ex H.B.K., *Pennisetum setaceum* (Forssk.) Chiov., *Ricinus communis* L., *Typha angustifolia* L.	[85,86,88,115,121,132,145,171,172,173,174,175,176,177,178,179,180]

## Data Availability

Not applicable.

## References

[1] Gaertner M., Den Breeyen A., Hui C., Richardson D.M. (2009). Impacts of alien plant invasions on species richness in Mediterranean-type ecosystems: A meta-analysis. Prog. Phys. Geogr..

[2] Le Maître D.C., Gaertner M., Marchante E., Ens E.-J., Holmes P.M., Pauchard A., O’Farrell P.J., Rogers A.M., Blanchard R., Blignaut J. (2011). Impacts of invasive Australian acacias: Implications for management and restoration. Divers. Distrib..

[3] Simberloff D. (2011). How common are invasion-induced ecosystem impacts?. Biol. Invasions.

[4] Foxcroft L.C., Pyšek P., Richardson D.M., Genovesi P., MacFadyen S. (2017). Plant invasion science in protected areas: Progress and priorities. Biol. Invasions.

[5] Shackleton R.T., Foxcroft L.C., Pyšek P., Wood L.E., Richardson D.M. (2020). Assessing biological invasions in protected areas after 30 years: Revisiting nature reserves targeted by the 1980s SCOPE programme. Biol. Conserv..

[6] Mack R.N., Lonsdale W.M. (2001). Humans as global plant dispersers: Getting more than we bargained for: Current introductions of species for aesthetic purposes present the largest single challenge for predicting which plant immigrants will become future pests. BioScience.

[7] Brundu G., Richardson D.M. (2016). Planted forests and invasive alien trees in Europe: A code for managing existing and future plantings to mitigate the risk of negative impacts from invasions. NeoBiota.

[8] Hulme P.E., Pyšek P., Jarošík V., Pergl J., Schaffner U., Vilà M. (2013). Bias and error in understanding plant invasion impacts. Trends Ecol. Evol..

[9] Milanović M., Knapp S., Pyšek P., Kühn I. (2020). Trait–environment relationships of plant species at different stages of the introduction process. NeoBiota.

[10] Dzikiti S., Ntshidi Z., Le Maitre D.C., Bugan R.D., Mazvimavi D., Schachtschneider K., Jovanovic N.Z., Pienaar H.H. (2017). Assessing water use by *Prosopis* invasions and *Vachellia karroo* trees: Implications for groundwater recovery following alien plant removal in an arid catchment in South Africa. For. Ecol. Manag..

[11] Mugnai M., Benesperi R., Viciani D., Ferretti G., Giunti M., Giannini F., Lazzaro L. (2022). Impacts of the invasive alien *Carpobrotus* spp. on coastal habitats on a Mediterranean island (Giglio Island, Central Italy). Plants.

[12] Radtke A., Ambraß S., Zerbe S., Tonon G., Fontana V., Ammer C. (2013). Traditional coppice forest management drives the invasion of *Ailanthus altissima* and *Robinia pseudoacacia* into deciduous forests. For. Ecol. Manag..

[13] Lorenzo P., González L., Ferrero V. (2021). Effect of plant origin and phenological stage on the allelopathic activity of the invasive species *Oxalis pes-caprae*. Am. J. Bot..

[14] Volakakis N., Kabourakis E., Rempelos L., Kiritsakis A., Leifert C. (2022). Effect of different cover crops, mass-trapping systems and environmental factors on invertebrate activity in table olive orchards—Results from field experiments in Crete, Greece. Agronomy.

[15] Pintor-Ibarra L.F., Rivera-Prado J.J., Ngangyo-Heya M., Rutiaga-Quiñones J.G. (2018). Evaluation of the chemical components of *Eichhornia crassipes* as an alternative raw material for pulp and paper. BioResources.

[16] Bakrim W.B., Ezzariai A., Karouach F., Sobeh M., Kibret M., Hafidi M., Kouisni L., Yasri A. (2022). *Eichhornia crassipes* (Mart.) Solms: A comprehensive review of its chemical composition, traditional use, and value-added products. Front. Pharmacol..

[17] Lorenzo P., Pazos-Malvido E., Rubido-Bará M., Reigosa M.J., González L. (2012). Invasion by the leguminous tree *Acacia dealbata* (Mimosaceae) reduces the native understorey plant species in different communities. Aust. J. Bot..

[18] Lorenzo P., Pereira C.S., Rodríguez-Echeverría S. (2013). Differential impact on soil microbes of allelopathic compounds released by the invasive *Acacia dealbata* Link. Soil Biol. Biochem..

[19] Rodríguez-Echeverría S., Afonso C., Correia M., Lorenzo P., Roiloa S.R. (2013). The effect of soil legacy on competition and invasion by *Acacia dealbata* Link. Plant Ecol..

[20] Lazzaro L., Giuliani C., Fabiani A., Agnelli A.E., Pastorelli R., Lagomarsino A., Benesperi R., Calamassi R., Foggi B. (2014). Soil and plant changing after invasion: The case of *Acacia dealbata* in a Mediterranean ecosystem. Sci. Total Environ..

[21] Keet J.H., Ellis A.G., Hui C., Nóvoa A., Le Roux J.J. (2021). Impacts of invasive Australian acacias on soil bacterial community composition, microbial enzymatic activities, and nutrient availability in Fynbos Soils. Microb. Ecol..

[22] Yapi T.S., O’Farrell P.J., Dziba L.E., Esler K.J. (2018). Alien tree invasion into a South African montane grassland ecosystem: Impact of *Acacia* species on rangeland condition and livestock carrying capacity. Int. J. Biodiv. Sci. Ecosys. Serv. Manag..

[23] van Wilgen B.W., Zengeya T.A., Richardson D.M. (2022). A review of the impacts of biological invasions in South Africa. Biol. Invasions.

[24] Marchante E., Kjøller A., Struwe S., Freitas H. (2008). Short-and long-term impacts of *Acacia longifolia* invasion on the belowground processes of a Mediterranean coastal dune ecosystem. Appl. Soil Ecol..

[25] Raghurama M., Sankaran M. (2022). Invasive nitrogen-fixing plants increase nitrogen availability and cycling rates in a montane tropical grassland. Plant Ecol..

[26] Le Maitre D.C., Blignaut J.N., Clulow A., Dzikiti S., Everson C.S., Grgens A.H.M., Gush M.B., van Wilgen B.W., Measey J., Richardson D.M., Wilson J.R., Zengeya T.A. (2020). Impacts of plant invasions on terrestrial water flows in South Africa. Biological Invasions in South Africa.

[27] Fletcher R.A., Brooks R.K., Lakoba V.T., Sharma G., Heminger A.R., Dickinson C.C., Barney J.N. (2019). Invasive plants negatively impact native, but not exotic, animals. Glob. Chang. Biol..

[28] Rodríguez J., Cordero-Rivera A., González L. (2021). Impacts of the invasive plant *Carpobrotus edulis* on herbivore communities on the Iberian Peninsula. Biol. Invasions.

[29] Rai P.K., Singh J.S. (2020). Invasive alien plant species: Their impact on environment, ecosystem services and human health. Ecol. Indic..

[30] Fantle-Lepczyk J.E., Haubrock P.J., Kramer A.M., Cuthbert R.N., Turbelin A.J., Crystal-Ornelas R., Diagne C., Courchamp F. (2022). Economic costs of biological invasions in the United States. Sci. Total Environ..

[31] Haubrock P.J., Turbelin A.J., Cuthbert R.N., Nóvoa A., Taylor N.G., Angulo E., Ballesteros-Mejia L., Bodey T.W., Capinha C., Diagne C. (2021). Economic costs of invasive alien species across Europe. NeoBiota.

[32] Cuthbert R.N., Diagne C., Hudgins E.J., Turbelin A., Ahmed D.A., Albert C., Bodey T.W., Briski E., Essl F., Haubrock P.J. (2022). Biological invasion costs reveal insufficient proactive management worldwide. Sci. Total Environ..

[33] Moodley D., Angulo E., Cuthbert R.N., Leung B., Turbelin A.J., Novoa A., Kourantidou M., Heringer G., Haubrock P.J., Renault D. (2022). Surprisingly high economic costs of biological invasions in protected areas. Biol. Invasions.

[34] Kourantidou M., Cuthbert R.N., Haubrock P.J., Novoa A., Taylor N.G., Leroy B., Capinha C., Renault D., Angulo E., Diagne C. (2021). Economic costs of invasive alien species in the Mediterranean basin. NeoBiota.

[35] Novoa A., Dehnen-Schmutz K., Fried J., Vimercati G. (2017). Does public awareness increase support for invasive species management? Promising evidence across taxa and landscape types. Biol. Invasions.

[36] Ricciardi A., Iacarella J.C., Aldridge D.C., Blackburn T.M., Carlton J.T., Catford J.A., Dick J.T.A., Hulme P., Jeschke J.M., Liebhold A.M. (2021). Four priority areas to advance invasion science in the face of rapid environmental change. Environ. Rev..

[37] Hussner A., Stiers I., Verhofstad M.J.J.M., Bakker E.S., Grutters B.M.C., Haury J., van Valkenburg J.L.C.H., Brundu G., Newman J., Clayton J.S. (2017). Management and control methods of invasive alien freshwater aquatic plants: A review. Aquat. Bot..

[38] Gala-Czekaj D., Synowiec A., Dąbkowska T. (2021). Self-renewal of invasive goldenrods (*Solidago* spp.) as a result of different mechanical management of fallow. Agronomy.

[39] Gaskin J.F., Espeland E., Johnson C.D., Larson D.L., Mangold J.M., McGee R.A., Milner C., Paudel S., Pearson D.E., Perkins L.B. (2021). Managing invasive plants on Great Plains grasslands: A discussion of current challenges. Rang. Ecol. Manag..

[40] Cohen O., Bar P., Gamliel A., Katan J., Kurzbaum E., Weber G., Schubert I., Riov J. (2019). Rain-based soil solarization for reducing the persistent seed banks of invasive plants in natural ecosystems–*Acacia saligna* as a model. Pest Manag. Sci..

[41] Jones P., Tummers J., Galib S., Woodford D., Hume J., Silva L., Braga R., Garcia de Leaniz C., Vitule J., Herder J. (2021). The use of barriers to limit the spread of aquatic invasive animal species: A global review. Front. Ecol. Evol..

[42] Souza-Alonso P., Lorenzo P., Rubido-Bará M., González L. (2013). Effectiveness of management strategies in *Acacia dealbata* Link invasion, native vegetation and soil microbial community responses. For. Ecol. Manag..

[43] Lazzaro L., Tondini E., Lombardi L., Giunti M. (2020). The eradication of *Carpobrotus* spp. in the sand-dune ecosystem at Sterpaia (Italy, Tuscany): Indications from a successful experience. Biologia.

[44] Núñez-González N., Rodríguez J., González L. (2021). Managing the invasive plant *Carpobrotus edulis*: Is mechanical control or specialized natural enemy more effective?. J. Environ. Manag..

[45] Muvengwi J., Mbiba M., Jimu L., Mureva A., Dodzo B. (2018). An assessment of the effectiveness of cut and ring barking as a method for control of invasive *Acacia mearnsii* in Nyanga National Park, Zimbabwe. For. Ecol. Manag..

[46] Sher A.A., El Waer H., González E., Anderson R., Henry A.L., Biedron R., Yue P. (2018). Native species recovery after reduction of an invasive tree by biological control with and without active removal. Ecol. Eng..

[47] Verbrugge L.N.H., de Hoop L., Aukema R., Beringen R., Creemers R.C.M., van Duinen G.A., Hollander H., de Hullu E., Scherpenisse M., Spikmans F. (2019). Lessons learned from rapid environmental risk assessments for prioritization of alien species using expert panels. J. Environ. Manag..

[48] Osunkoya O.O., Froese J.G., Nicol S. (2019). Management feasibility of established invasive plant species in Queensland, Australia: A stakeholders’ perspective. J. Environ. Manag..

[49] Delbart E., Mahy G., Weickmans B., Henriet F., Crémer S., Pieret N., Vanderhoeven S., Monty A. (2012). Can land managers control Japanese knotweed? Lessons from control tests in Belgium. Environ. Manag..

[50] Frelich M., Bzdęga K. (2014). Management of invasive plant species in the valley of the River Ślepiotka in Katowice–The example of the REURIS project. Environ. Socio-Econ. Stud..

[51] Froeschlin N., Privett S.D., Richardson D.M., Gaertner M. (2022). Fynbos vegetation recovery twelve years after removal of invasive *Eucalyptus* trees. S. Afr. J. Bot..

[52] Duarte L.N., Marchante E., Marchante H. (2023). Managing an invasive tree in coastal dunes: The importance of follow-up treatments to improve the recovery of protected habitats. Front. Environ. Sci..

[53] Dana E.D., García-de-Lomas J., Verloove F., Vilà M. (2019). Common deficiencies of actions for managing invasive alien species: A decision-support checklist. NeoBiota.

[54] Rani L., Thapa K., Kanojia N., Sharma N., Singh S., Grewal A.S., Srivastav A.L., Kaushal J. (2020). An extensive review on the consequences of chemical pesticides on human health and environment. J. Clean. Prod..

[55] Choudri B., Charabi Y., Ahmed M. (2018). Pesticides and herbicides. Water Environ. Res..

[56] Chauvel B., Fried G., Follak S., Chapman D., Kulakova Y., Le Bourgeois T., Marisavlievic D., Monty A., Rossi J.-P., Starfinger U. (2021). Monographs on invasive plants in Europe N° 5: *Ambrosia trifida* L.. Bot. Lett..

[57] Brodeur J. (2012). Host specificity in biological control: Insights from opportunistic pathogens. Evol. Appl..

[58] Corbin J.D., Wolford M., Zimmerman C.L., Quirion B. (2017). Assessing feasibility in invasive plant management: A retrospective analysis of garlic mustard (*Alliaria petiolata*) control. Restor. Ecol..

[59] Perry G.L., Moloney K.A., Etherington T.R. (2017). Using network connectivity to prioritise sites for the control of invasive species. J. Appl. Ecol..

[60] Van Wilgen B.W., Wannenburgh A. (2016). Co-facilitating invasive species control, water conservation and poverty relief: Achievements and challenges in South Africa’s Working for Water programme. Curr. Opin. Environ. Sustain..

[61] Novoa A., Kaplan H., Kumschick S., Wilson J.R., Richardson D.M. (2015). Soft touch or heavy hand? Legislative approaches for preventing invasions: Insights from cacti in South Africa. Invasive Plant Sci. Manag..

[62] Epanchin-Niell R., Thompson A.L., Treakle T. (2021). Public contributions to early detection of new invasive pests. Conserv. Sci. Pract..

[63] Price-Jones V., Brown P.M.J., Adriaens T., Tricarico E., Farrow R.A., Cardoso A.C., Gervasini E., Groom Q., Reyserhove L., Schade S. (2022). Eyes on the aliens: Citizen science contributes to research, policy and management of biological invasions in Europe. NeoBiota.

[64] Encarnação J., Teodósio M.A., Morais P. (2021). Citizen science and biological invasions: A review. Front. Environ. Sci..

[65] Anđelković A.A., Handley L.L., Marchante E., Adriaens T., Brown P.M.J., Tricarico E., Verbrugge L.N.H. (2022). A review of volunteers’ motivations to monitor and control invasive alien species. NeoBiota.

[66] Jubase N., Shackleton R.T., Measey J. (2021). Motivations and contributions of volunteer groups in the management of invasive alien plants in South Africa’s Western Cape province. Bothalia Afr. Biodivers. Conserv..

[67] Ulm F., Estorninho M., de Jesus J.G., de Sousa Prado M.G., Cruz C., Máguas C. (2022). From a lose–lose to a win–win situation: User-friendly biomass models for *Acacia longifolia* to aid research, management and valorisation. Plants.

[68] Panetta F.D., O’Loughlin L.S., Gooden B. (2019). Identifying thresholds and ceiling in plant community recovery for optimal management of widespread weeds. NeoBiota.

[69] Brito L.M., Mourão I., Coutinho J., Smith S.R. (2015). Co-composting of invasive *Acacia longifolia* with pine bark for horticultural use. Environ. Technol..

[70] Brito L.M., Reis M., Mourão I., Coutinho J. (2015). Use of acacia waste compost as an alternative component for horticultural substrates. Commun. Soil Sci. Plant Anal..

[71] Mesa F., Torres J., Sierra O., Escobedo F.J. (2017). Enhanced production of compost from Andean wetland biomass using a bioreactor and photovoltaic system. Biomass Bioenergy.

[72] Mulvaney M.J., Wood C.W., Balkcom K.S., Kemble J., Shannon D.A. (2017). No-till with high biomass cover crops and invasive legume mulches increased total soil carbon after three years of collard production. Agroecol. Sustain. Food Syst..

[73] Cvejić R., Klages S., Pintar M., Resman L., Slatnar A., Mihelič R. (2021). Invasive plants in support of urban farming: Fermentation-based organic fertilizer from Japanese Knotweed. Agronomy.

[74] Liu H., Zhao Q., Cheng Y. (2022). Composted invasive plant *Ageratina adenophora* enhanced barley (*Hordeum vulgare*) growth and soil conditions. PLoS ONE.

[75] Vyankatrao N.P. (2017). Conversion of *Parthenium hystorophorus* L. weed to compost and vermicompost. Biosci. Discov..

[76] Lorenzo P., Álvarez-Iglesias L., González L., Revilla P. (2022). Assessment of *Acacia dealbata* as green manure and weed control for maize crop. Renew. Agric. Food Syst..

[77] Alami E., Karimi M., Chalavi V. (2021). Investigation of compost and vermicompost of water hyacinth as growing media for Lily (*Longiflorum* × *Asiatic*). Int. J. Hortic. Sci. Technol..

[78] Islam M.N., Rahman F., Papri S.A., Faruk M.O., Das A.K., Adhikary N., Debrot A.O., Ahsan M.N. (2021). Water hyacinth (*Eichhornia crassipes* (Mart.) Solms.) as an alternative raw material for the production of bio-compost and handmade paper. J. Environ. Manag..

[79] Gosal M., Rayer D., Gedoan S. (2022). The effect of water hyacinth (*Eichhornia crassipes*) organic fertilizer on the vegetative growth of Manado strain yellow maize (*Zea mays* L.). World J. Adv. Res. Rev..

[80] Ogutu P.A. (2019). Vermicomposting water hyacinth: Turning Fisherman’s Nightmare into Farmer’s Fortune. Int. J. Res. Innov. Appl. Sci..

[81] Feng Q., Wang B., Chen M., Wu P., Lee X., Xing Y. (2021). Invasive plants as potential sustainable feedstocks for biochar production and multiple applications: A review. Resour. Conserv. Recycl..

[82] Patwa D., Muigai H.H., Ravi K., Sreedeep S., Kalita P. (2022). A novel application of biochar produced from invasive weeds and industrial waste in thermal backfill for crude oil industries. Waste Biomass Valorization.

[83] Kleinschroth F., Winton R.S., Calamita E., Niggemann F., Botter M., Wehrli B., Ghazoul J. (2021). Living with floating vegetation invasions. Ambio.

[84] Arutselvy B., Rajeswari G., Jacob S. (2021). Sequential valorization strategies for dairy wastewater and water hyacinth to produce fuel and fertilizer. J. Food Process Eng..

[85] Harun I., Pushiri H., Amirul-Aiman A.J., Zulkeflee Z. (2021). Invasive water hyacinth: Ecology, impacts and prospects for the rural economy. Plants.

[86] Ilo O.P., Simatele M.D., Nkomo S.P.L., Mkhize N.M., Prabhu N.G. (2020). The benefits of water hyacinth (*Eichhornia crassipes*) for Southern Africa: A review. Sustainability.

[87] Sladonja B., Sušek M., Guillermic J. (2015). Review on invasive tree of heaven (*Ailanthus altissima* (Mill.) Swingle) conflicting values: Assessment of its ecosystem services and potential biological threat. Environ. Manag..

[88] López-Hortas L., Rodríguez-González I., Díaz-Reinoso B., Torres M.D., Moure A., Domínguez H. (2021). Tools for a multiproduct biorefinery of *Acacia dealbata* biomass. Ind. Crops Prod..

[89] Souza-Alonso P., Puig C.G., Pedrol N., Freitas H., Rodríguez-Echeverría S., Lorenzo P. (2020). Exploring the use of residues from the invasive *Acacia* sp. for weed control. Renew. Agric. Food Syst..

[90] Brito L.M., Mourão I., Coutinho J., Smith S. (2013). Composting for management and resource recovery of invasive *Acacia* species. Waste Manag. Res..

[91] Adam Y., Sershen R.S. (2016). Maize and pea germination and seedling growth responses to compost generated from biowaste of selected invasive alien plant species. Compost Sci. Util..

[92] Chemetova C., Ribeiro H., Fabião A., Gominho J. (2020). Towards sustainable valorisation of *Acacia melanoxylon* biomass: Characterization of mature and juvenile plant tissues. Environ. Res..

[93] Yan S.H., Song W., Guo J.Y. (2017). Advances in management and utilization of invasive water hyacinth (*Eichhornia crassipes*) in aquatic ecosystems–a review. Crit. Rev. Biotechnol..

[94] Kumwimba M.N., Dzakpasu M., Li X. (2020). Potential of invasive watermilfoil (*Myriophyllum* spp.) to remediate eutrophic waterbodies with organic and inorganic pollutants. J. Environ. Manag..

[95] Quintela-Sabarís C., Mendes L.A., Domínguez J. (2022). Vermicomposting as a sustainable option for managing biomass of the invasive tree *Acacia dealbata* Link. Sustainability.

[96] Balachandar R., Biruntha M., Yuvaraj A., Thangaraj R., Subbaiya R., Govarthanan M., Kumar P., Karmegam N. (2021). Earthworm intervened nutrient recovery and greener production of vermicompost from *Ipomoea staphylina*–An invasive weed with emerging environmental challenges. Chemosphere.

[97] Abbas A.M., Novak S.J., Fictor M., Mostafa Y.S., Alamri S.A., Alrumman S.A., Taher M.A., Hashem M., Khalaphallah R. (2022). Initial in vitro assessment of the antifungal activity of aqueous extracts from three invasive plant species. Agriculture.

[98] Jiao Y., Li Y., Yuan L., Huang J. (2021). Allelopathy of uncomposted and composted invasive aster (*Ageratina adenophora*) on ryegrass. J. Hazard. Mater..

[99] Liua H., Wangc Y., Zhaoa Q. (2022). Converting invasive aster (*Ageratina adenophora* L.) into organic fertilizer source. ScienceAsia.

[100] Li P., Chang Q., Wang C., Cao J., Zheng W. (2014). Composting of aerial parts of crofton weed (*Eupatorium adenophorum* Spreng), the top invasive plant in southwest China. Compost Sci. Util..

[101] Ulm F., Avelar D., Hobson P., Penha-Lopes G., Dias T., Máguas C., Cruz C. (2019). Sustainable urban agriculture using compost and an open-pollinated maize variety. J. Clean. Prod..

[102] Devi C., Khwairakpam M. (2021). Management of invasive weed *Parthenium hysterophorus* through vermicomposting using a polyculture of *Eisenia fetida* and *Eudrilus eugeniae*. Environ. Sci. Pollut. Res..

[103] Hussain N., Abbasi T., Abbasi S.A. (2016). Vermicomposting transforms allelopathic parthenium into a benign organic fertilizer. J. Environ. Manag..

[104] Benelli G., Pavela R., Cianfaglione K., Nagy D.U., Canale A., Maggi F. (2019). Evaluation of two invasive plant invaders in Europe (*Solidago canadensis* and *Solidago gigantea*) as possible sources of botanical insecticides. J. Pest Sci..

[105] Lorenzo P., Reboredo-Durán J., Múñoz L., González L., Freitas H., Rodríguez-Echeverría S. (2016). Inconsistency in the detection of phytotoxic effects: A test with *Acacia dealbata* extracts using two different methods. Phytochem. Lett..

[106] Lorenzo P., Souza-Alonso P., Guisande-Collazo A., Freitas H. (2019). Influence of *Acacia dealbata* Link bark extracts on the growth of *Allium cepa* L. plants under high salinity conditions. J. Sci. Food Agric..

[107] Lorenzo P., Reboredo-Durán J., Muñoz L., Freitas H., González L. (2020). Herbicidal properties of the commercial formulation of methyl cinnamate, a natural compound in the invasive silver wattle (*Acacia dealbata*). Weed Sci..

[108] Quinty V., Colas C., Nasreddine R., Nehmé R., Piot C., Draye M., Destandau E., Da Silva D., Chatel G. (2022). Screening and evaluation of dermo-cosmetic activities of the invasive plant species *Polygonum cuspidatum*. Plants.

[109] Oliveira C.S., Moreira P., Resende J., Cruz M.T., Pereira C.M., Silva A.M., Santos S.A.O., Silvestre A.J. (2020). Characterization and cytotoxicity assessment of the lipophilic fractions of different morphological parts of *Acacia dealbata*. Int. J. Mol. Sci..

[110] Míguez C., Cancela Á., Sánchez Á., Álvarez X. (2022). Possibilities for exploitation of invasive species, *Arundo donax* L., as a source of phenol compounds. Waste Biomass Valor..

[111] Okyere S.K., Wen J., Cui Y., Xie L., Gao P., Wang J., Wang S., Hu Y. (2021). Toxic mechanisms and pharmacological properties of euptox A, a toxic monomer from *A*. adenophora. Fitoterapia.

[112] Peter A., Žlabur J.Š., Šurić J., Voća S., Purgar D.D., Pezo L., Voća N. (2021). Invasive plant species biomass—Evaluation of functional value. Molecules.

[113] Correia R., Duarte M.P., Maurício E.M., Brinco J., Quintela J.C., da Silva M.G., Gonçalves M. (2022). Chemical and functional characterization of extracts from leaves and twigs of *Acacia dealbata*. Processes.

[114] Paula V., Pedro S.I., Campos M.G., Delgado T., Estevinho L.M., Anjos O. (2022). Special bioactivities of phenolics from *Acacia dealbata* L. with potential for dementia, diabetes and antimicrobial Treatments. Appl. Sci..

[115] Kaur A., Batish D.R., Kaur S., Chauhan B.S. (2021). An overview of the characteristics and potential of *Calotropis procera* from botanical, ecological, and economic perspectives. Front. Plant Sci..

[116] Casas M.P., López-Hortas L., Díaz-Reinoso B., Moure A., Domínguez H. (2021). Supercritical CO_2_ extracts from *Acacia dealbata* flowers. J. Supercrit. Fluids.

[117] Ponticelli M., Lela L., Russo D., Faraone I., Sinisgalli C., Mustapha M.B., Esposito G., Jannet H.B., Costantino V., Milella L. (2022). *Dittrichia graveolens* (L.) Greuter, a rapidly spreading invasive plant: Chemistry and bioactivity. Molecules.

[118] Arsene M.M.J., Viktorovna P.I., Mikhaïlovitch M.K., Davares A.K.L., Parfait K., Rehailia M., Nikolayevich S.A., Stefanovna G.V., Sarra S., Sulikoevich K.Z. (2022). In vitro antimicrobial activity, antibioresistance reversal properties, and toxicity screen of ethanolic extracts of *Heracleum mantegazzianum* Sommier and Levier (giant hogweed), *Centaurea jacea* L. (brown knapweed), and *Chenopodium album* L. (Pigweed): Three invasive plants. Open Vet. J..

[119] Kim G.J., Park S., Kim E., Kwon H., Park H.J., Nam J.W., Roh S.S., Choi H. (2021). Antioxidant, pancreatic lipase inhibitory, and tyrosinase inhibitory activities of extracts of the invasive plant *Spartina anglica* (Cord-Grass). Antioxidants.

[120] Yildiz S., Gurgen A., Can Z., Tabbouche S.A., Kilic A.O. (2018). Some bioactive properties of *Acacia dealbata* extracts and their potential utilization in wood protection. Drewno.

[121] Olayiwola H.O., Amiandamhen S.O., Meincken M., Tyhoda L. (2021). Investigating the suitability of fly ash/metakaolin-based geopolymers reinforced with South African alien invasive wood and sugarcane bagasse residues for use in outdoor conditions. Eur. J. Wood Wood Prod..

[122] Rodrigues V.H., de Melo M.M., Portugal I., Silva C.M. (2021). Extraction of added-value triterpenoids from *Acacia dealbata* leaves using supercritical fluid extraction. Processes.

[123] Rodrigues V.H., de Melo M.M., Portugal I., Silva C.M. (2021). Lupane-type triterpenoids from *Acacia dealbata* bark extracted by different methods. Ind. Crops Prod..

[124] Míguez C., Cancela A., Álvarez X., Sánchez A. (2022). The reuse of bio-waste from the invasive species *Tradescantia fluminensis* as a source of phenolic compounds. J. Clean. Prod..

[125] Borges A., José H., Homem V., Simões M. (2020). Comparison of techniques and solvents on the antimicrobial and antioxidant potential of extracts from *Acacia dealbata* and *Olea europaea*. Antibiotics.

[126] Neiva D.M., Luís A., Gominho J., Domingues F., Duarte A.P., Pereira H. (2020). Bark residues valorization potential regarding antioxidant and antimicrobial extracts. Wood Sci. Technol..

[127] Marinas I.C., Oprea E., Geana E.I., Tutunaru O., Pircalabioru G.G., Zgura I., Chifiriuc M.C. (2020). Valorization of *Gleditsia triacanthos* invasive plant cellulose microfibers and phenolic compounds for obtaining multi-functional wound dressings with antimicrobial and antioxidant properties. Int. J. Mol. Sci..

[128] Yáñez R., Gómez B., Martínez M., Gullón B., Alonso J.L. (2014). Valorization of an invasive woody species, *Acacia dealbata*, by means of Ionic liquid pretreatment and enzymatic hydrolysis. J. Chem. Technol. Biotechnol..

[129] Iyer A., Bestwick C.S., Duncan S.H., Russell W.R. (2021). Invasive plants are a valuable alternate protein source and can contribute to meeting climate change targets. Front. Sustain. Food Syst..

[130] Iyer A., Guerrier L., Leveque S., Bestwick C.S., Duncan S.H., Russell W.R. (2022). High throughput method development and optimised production of leaf protein concentrates with potential to support the agri-industry. J. Food Meas. Characterizat..

[131] Sowndhararajan K., Santhanam R., Hong S., Jhoo J.W., Kim S. (2016). Suppressive effects of acetone extract from the stem bark of three *Acacia* species on nitric oxide production in lipopolysaccharide-stimulated RAW 264.7 macrophage cells. Asian Pac. J. Trop. Biomed..

[132] Martínez-Espinosa J.C., Ramírez-Morales M.A., Carrera-Cerritos R. (2022). Silver nanoparticles synthesized using *Eichhornia crassipes* extract from Yuriria lagoon, and the perspective for application as antimicrobial agent. Crystals.

[133] Jayaweera M.W., Dilhani J.A., Kularatne R.K., Wijeyekoon S.L. (2007). Biogas production from water hyacinth (Eichhornia crassipes (Mart.) Solms) grown under different nitrogen concentrations. J. Environ. Sci. Health Part A.

[134] Van Meerbeek K., Appels L., Dewil R., Calmeyn A., Lemmens P., Muys B., Hermy M. (2015). Biomass of invasive plant species as a potential feedstock for bioenergy production. Biofuels Bioprod. Biorefining.

[135] Ferreira S., Gil N., Queiroz J.A., Duarte A.P., Domingues F.C. (2011). An evaluation of the potential of *Acacia dealbata* as raw material for bioethanol production. Bioresour. Technol..

[136] Zhang Q., Weng C., Huang H., Achal V., Wang D. (2016). Optimization of bioethanol production using whole plant of water hyacinth as substrate in simultaneous saccharification and fermentation process. Front. Microbiol..

[137] Carlini M., Castellucci S., Mennun A. (2018). Water hyacinth biomass: Chemical and thermal pre-treatment for energetic utilization in anaerobic digestion process. Energy Procedia.

[138] Alves J.L.F., da Silva J.C.G., da Silva Filho V.F., Alves R.F., de Araujo Galdino W.V., De Sena R.F. (2019). Kinetics and thermodynamics parameters evaluation of pyrolysis of invasive aquatic macrophytes to determine their bioenergy potentials. Biomass Bioenergy.

[139] Nunes L.J., Raposo M.A., Meireles C.I., Pinto Gomes C.J., Ribeiro N.M.A. (2020). Control of invasive forest species through the creation of a value chain: *Acacia dealbata* biomass recovery. Environments.

[140] Van Tran G., Unpaprom Y., Ramaraj R. (2020). Methane productivity evaluation of an invasive wetland plant, common reed. Biomass Conver. Biorefinery.

[141] da Costa R.M., Bosch M., Simister R., Gomez L.D., Canhoto J.M., Batista de Carvalho L.A. (2022). Valorisation potential of invasive *Acacia dealbata*, *A*. longifolia and A. melanoxylon from land clearings. Molecules.

[142] Liao R., Gao B., Fang J. (2013). Invasive plants as feedstock for biochar and bioenergy production. Bioresour. Technol..

[143] Albaugh T.J., Rubilar R.A., Maier C.A., Acuna E.A., Cook R.L. (2017). Biomass and nutrient mass of *Acacia dealbata* and *Eucalyptus globulus* bioenergy plantations. Biomass Bioenergy.

[144] Stafford W., Blignaut J. (2017). Reducing landscape restoration costs: Feasibility of generating electricity from invasive alien plant biomass on the Agulhas Plain, South Africa. Ecosyst. Serv..

[145] Ngorima A., Shackleton C.M. (2019). Livelihood benefits and costs from an invasive alien tree (*Acacia dealbata*) to rural communities in the Eastern Cape, South Africa. J. Environ. Manag..

[146] Carson B.D., Lishawa S.C., Tuchman N.C., Monks A.M., Lawrence B.A., Albert D.A. (2018). Harvesting invasive plants to reduce nutrient loads and produce bioenergy: An assessment of Great Lakes coastal wetlands. Ecosphere.

[147] Awasthi M., Kaur J., Rana S. (2013). Bioethanol production through water hyacinth, *Eichhornia crassipes* via optimization of the pretreatment conditions. Int. J. Emerg. Technol. Adv. Eng..

[148] Ruan T., Zeng R., Yin X.Y., Zhang S.X., Yang Z.H. (2016). Water hyacinth (*Eichhornia crassipes*) biomass as a biofuel feedstock by enzymatic hydrolysis. BioResources.

[149] Łapczyńska-Kordon B., Ślipek Z., Słomka-Polonis K., Styks J., Hebda T., Francik S. (2022). Physicochemical properties of biochar produced from goldenrod plants. Materials.

[150] Raj F.R.M.S., Boopathi G., Kalpana D., Jaya N.V., Pandurangan A. (2022). Sustainable development through restoration of *Prosopis juliflora* species into activated carbon as electrode material for supercapacitors. Diam. Relat. Mater..

[151] Yang L., Deng Y., Shu Z., Chen Q., Yang H., Tan X. (2022). Application of invasive plants as biochar precursors in the field of environment and energy storage. Front. Environ. Sci..

[152] Muñoz C., Mendonça R., Baeza J., Berlin A., Saddler J., Freer J. (2007). Bioethanol production from bio-organosolv pulps of *Pinus radiata* and *Acacia dealbata*. J. Chem. Technol. Biotechnol. Int. Res. Process Environ. Clean Technol..

[153] Nguyen X.C., Nguyen T.T.H., Nguyen T.H.C., Le Q.V., Vo T.Y.B., Tran T.C.P., La D.D., Kumar G., Nguyen V.K., Chang S.W. (2021). Sustainable carbonaceous biochar adsorbents derived from agro-wastes and invasive plants for cation dye adsorption from water. Chemosphere.

[154] Tham H.T., Udén P. (2013). Effect of water hyacinth (*Eichhornia crassipes*) silage on intake and nutrient digestibility in cattle fed rice straw and cottonseed cake. Asian-Australas. J. Anim. Sci..

[155] Korkut A.Y., Gunes A., Kop A., Cakar H., Akat O., Guney M.A., Ozkul B., Koru E., Suzer C., Cirik S. (2016). Preliminary study for utilization of some invasive aquatic plants as raw material for aquaculture feeds. Fresenius Environ. Bull..

[156] Moselhy M.A., Borba J.P., Borba A.E. (2022). Production of high-quality silage from invasive plants plus agro-industrial by-products with or without bacterial inoculation. Biocata. Agric. Biotechnol..

[157] Pratiwi D.Y., Andhikawati A. (2021). Utilization of water hyacinth (*Eichhornia crassipes*) as fish feed ingredient. Asian J. Fish. Aquat. Res..

[158] Teixeira A.R., Jorge N., Fernandes J.R., Lucas M.S., Peres J.A. (2022). Textile dye removal by *Acacia dealbata* link. pollen adsorption combined with UV-A/NTA/Fenton process. Top. Catal..

[159] Carneiro M.T., Barros A.Z.B., Morais A.I.S., Carvalho Melo A.L.F., Bezerra R.D.S., Osajima J.A., Silva-Filho E.C. (2022). Application of water hyacinth biomass (*Eichhornia crassipes*) as an adsorbent for methylene blue dye from aqueous medium: Kinetic and isothermal study. Polymers.

[160] Zhang L., Cheng H., Pan D., Wu Y., Ji R., Li W., Jiang X., Han J. (2021). One-pot pyrolysis of a typical invasive plant into nitrogen-doped biochars for efficient sorption of phthalate esters from aqueous solution. Chemosphere.

[161] Almeida R., Cisneros F., Mendes C.V., Carvalho M.G.V., Rasteiro M.G., Gamelas J.A. (2021). Valorisation of invasive plant species in the production of polyelectrolytes. Ind. Crops Prod..

[162] Jorge N., Teixeira A.R., Lucas M.S., Peres J.A. (2022). Agro-industrial wastewater treatment with *Acacia dealbata* coagulation/flocculation and photo-Fenton-based processes. Recycling.

[163] Peng H., Wang Y., Tan T.L., Chen Z. (2020). Exploring the phytoremediation potential of water hyacinth by FTIR Spectroscopy and ICP-OES for treatment of heavy metal contaminated water. Int. J. Phytoremediation.

[164] Panneerselvam B., Priya K.S. (2021). Phytoremediation potential of water hyacinth in heavy metal removal in chromium and lead contaminated water. Int. J. Environ. Anal. Chem..

[165] Saha P., Shinde O., Sarkar S. (2017). Phytoremediation of industrial mines wastewater using water hyacinth. Int. J. Phytoremediation.

[166] Kavčič U., Karlovits I. (2020). The influence of process parameters of screen-printed invasive plant paper electrodes on cyclic voltammetry. Nord. Pulp Paper Res. J..

[167] Karlovits I., Lavrič G., Kavčič U., Zorić V. (2021). Electrophotography toner adhesion on agro-industrial residue and invasive plant papers. J. Adhes. Sci. Technol..

[168] Karlovits I., Kavčič U. (2022). Flexo printability of agro and invasive papers. Cellulose.

[169] Starešinič M., Boh Podgornik B., Javoršek D., Leskovšek M., Možina K. (2021). Fibers obtained from invasive alien plant species as a base material for paper production. Forests.

[170] Kapun T., Zule J., Fabjan E., Hočevar B., Grilc M., Likozar B. (2022). Engineered invasive plant cellulose fibers as resources for papermaking. Eur. J. Wood Wood Prod..

[171] Ranesi A., Faria P., Correia R., Freire M.T., Veiga R., Gonçalves M. (2022). Gypsum mortars with *Acacia dealbata* biomass waste additions: Effect of different fractions and contents. Buildings.

[172] Ortega Z., Romero F., Paz R., Suárez L., Benítez A.N., Marrero M.D. (2021). Valorization of invasive plants from Macaronesia as filler materials in the production of natural fiber composites by rotational molding. Polym..

[173] Portela-Grandío A., Peleteiro S., Yáñez R., Romaní A. (2021). Integral valorization of *Acacia dealbata* wood in organic medium catalyzed by an acidic ionic liquid. Bioresour. Technol..

[174] Rani B.S.J., Venkatachalam S. (2022). A neoteric approach for the complete valorization of *Typha angustifolia* leaf biomass: A drive towards environmental sustainability. J. Environ. Manag..

[175] Neiva D.M., Rencoret J., Marques G., Gutiérrez A., Gominho J., Pereira H., Del Río J.C. (2020). Lignin from tree barks: Chemical structure and valorization. ChemSusChem.

[176] Lim C.J., Arumugam M., Lim C.K., Ee G.C.L. (2018). Mercerizing extraction and physicochemical characterizations of lignocellulosic fiber from the leaf waste of *Mikania micrantha* Kunth ex HBK. J. Nat. Fibers.

[177] Čuk N., Šala M., Gorjanc M. (2021). Development of antibacterial and UV protective cotton fabrics using plant food waste and alien invasive plant extracts as reducing agents for the in-situ synthesis of silver nanoparticles. Cellulose.

[178] Arana-Cuenca A., Tovar-Jiménez X., Favela-Torres E., Perraud-Gaime I., González-Becerra A.E., Martínez A., Moss-Acosta C.L., Mercado-Flores Y., Téllez-Jurado A. (2019). Use of water hyacinth as a substrate for the production of filamentous fungal hydrolytic enzymes in solid-state fermentation. 3 Biotech.

[179] Linhares T., de Amorim M.T.P. (2017). LCA of textile dyeing with *Acacia dealbata* tree bark: A case study research. Procedia Eng..

[180] Lock Toy Ki Y., Garcia A., Pelissier F., Olszewski T.K., Babst-Kostecka A., Legrand Y.M., Grison C. (2022). Mechanochemistry and eco-bases for sustainable Michael addition reactions. Molecules.

[181] Shackleton R.T., Vimercati G., Probert A.F., Bacher S., Kull C.A., Novoa A. (2022). Consensus and controversy in the discipline of invasion science. Conserv. Biol..

[182] Dehnen-Schmutz K., Novoa A., Clements D.R., Upadhyaya M.K., Joshi S., Shrestha A. (2022). Advances in the management of invasive plants. Global Plant Invasions.

[183] Suárez L., Díaz T.E., Benavente-Ferraces I., Plaza C., Almeida M., Centeno T.A. (2022). Hydrothermal treatment as a complementary tool to control the invasive Pampas grass (*Cortaderia selloana*). Sci. Total Environ..

[184] Correia R., Quintela J.C., Duarte M.P., Gonçalves M. (2020). Insights for the valorization of biomass from Portuguese invasive *acacia* spp. in a biorefinery perspective. Forests.

[185] Nunes L.J.R., Rodrigues A.M., Loureiro L.M.E.F., Sá L.C.R., Matias J.C.O. (2021). Energy recovery from invasive species: Creation of value chains to promote control and eradication. Recycling.

[186] Vrabič-Brodnjak U., Možina K. (2022). Invasive alien plant species for use in paper and packaging materials. Fibers.

[187] Mudavanhu S., Blignaut J., Nkambule N., Morokong T., Vundla T. (2016). A cost-benefit analysis of using Rooikrans as biomass feedstock for electricity generation: A case study of the De Hoop nature reserve, South Africa. S. Afr. J. Econ. Manag. Sci..

[188] Melane M., Ham C., Meincken M. (2017). Characteristics of selected non-woody invasive alien plants in South Africa and an evaluation of their potential for electricity generation. J. Energy S. Afr..

[189] Valen M.A. (2017). Economic opportunities for biomass harvest of invasive giant reed (*Arundo donax* L.) in Southern California as feedstock for the pulp and paper industry. Master’s Thesis.

